# EnzymeMap: curation, validation and data-driven prediction of enzymatic reactions

**DOI:** 10.1039/d3sc02048g

**Published:** 2023-11-22

**Authors:** Esther Heid, Daniel Probst, William H. Green, Georg K. H. Madsen

**Affiliations:** a Institute of Materials Chemistry, TU Wien 1060 Vienna Austria esther.heid@tuwien.ac.at; b Department of Chemical Engineering, Massachusetts Institute of Technology Cambridge Massachusetts 02139 USA; c IBM Research Europe CH-8803 Rüschlikon Switzerland

## Abstract

Enzymatic reactions are an ecofriendly, selective, and versatile addition, sometimes even alternative to organic reactions for the synthesis of chemical compounds such as pharmaceuticals or fine chemicals. To identify suitable reactions, computational models to predict the activity of enzymes on non-native substrates, to perform retrosynthetic pathway searches, or to predict the outcomes of reactions including regio- and stereoselectivity are becoming increasingly important. However, current approaches are substantially hindered by the limited amount of available data, especially if balanced and atom mapped reactions are needed and if the models feature machine learning components. We therefore constructed a high-quality dataset (EnzymeMap) by developing a large set of correction and validation algorithms for recorded reactions in the literature and showcase its significant positive impact on machine learning models of retrosynthesis, forward prediction, and regioselectivity prediction, outperforming previous approaches by a large margin. Our dataset allows for deep learning models of enzymatic reactions with unprecedented accuracy, and is freely available online.

## Abstract 2

New curation and atom-mapping routine leading to large database of enzymatic reactions boosts performance of deep learning models.

## Graphical abstract

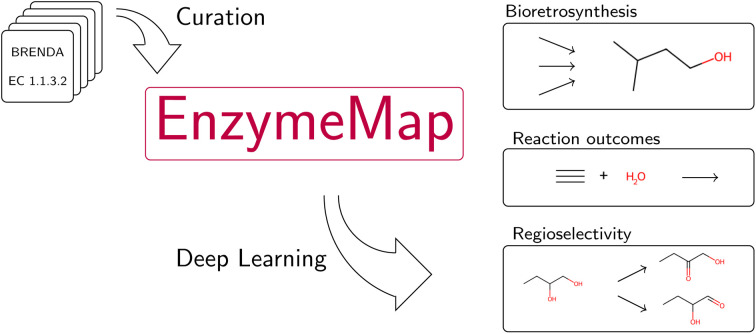

## 1 Introduction

Biocatalytic transformations are becoming increasingly important, attractive, and accessible for the synthesis of pharmaceuticals or fine chemicals.^1–11^ Recently, enzymatic synthesis routes to chemicals such as 1,4-butanediol,^12^ branched chain higher alcohols,^13^ or complex natural products such as the investigational HIV treatment islatravir,^14^ or the investigational antiviral agent molnupravir^15^ were developed. This interest is largely owed to the high chemo-, stereo-, and regioselectivity of enzymes,^2–5,16–18^ their applicability in mild reaction conditions and aqueous media, as well as their compatibility making the combination of several reaction steps in a single reaction vessel possible.^3,17,19^

Since enzymes can exhibit both substrate and reaction promiscuity,^20^*i.e.* can act on non-native substrates or catalyze new reactions, the toolbox of possible biocatalytic transformations extends well beyond reactions seen in nature. Low catalytic activity may be increased by directed evolution.^21–25^ Furthermore, computer-aided *de novo* design of enzymes has been reported for selected transformations,^26^ opening up the possibility to design an enzyme for a given task. Enzymatic reactions and cascades thus provide a promising eco-friendly alternative to conventional synthesis pathways for a large range of compounds.

However, compared to organic synthesis, where a huge variety of computational tools based on heuristics and machine learning aid the design of synthesis plans,^27–37^ enzymatic synthesis tools are less common and often limited in their applicability and accuracy. Existing tools for bioretrosynthesis planning,^38–44^ enzyme selection,^45,46^ and reaction rule extraction or scoring^47–49^ are often restricted by the available data, both concerning the amount and the quality of reported reactions in databases. Especially deep-learning-based approaches such as reinforcement learning models,^39^ or transformer models^43^ rely on large amounts of high-quality reaction data, which is easily obtainable for organic reactions, but not for enzymatic reactions. High-quality, curated datasets such as RHEA^50^ usually only report one or a few reactions per enzyme class and are thus small, whereas larger, uncurated databases such as BRENDA^51^ pose problems to resolve the provided substrate names, and may contain unbalanced or erroneous reactions. In addition, stereochemical aspects of enzymatic reactions are often disregarded, due to missing or erroneous entries in many databases. This is a major limitation of current approaches, since enzymatic reactions are favored especially for products containing multiple stereocenters, which can be difficult to obtain *via* organic reactions. Furthermore, atom mappings of enzymatic reactions in current databases are often not available, and can be tedious and error-prone to obtain, especially for biochemical transformations. Datasets of enzymatic reactions that include correct stereoinformation and are atom mapped, balanced, validated, diverse, and sufficiently large for data-driven deep learning applications are currently not available, severely hindering the development of new tools and models to design enzymatic synthesis pathways. In a sense, the current paradigm of data-driven research falls short in the field of enzymatic synthesis planning due to the limited size and quality of the available data. We therefore propose to instead follow a research-driven data approach, where the data necessary for new developments is identified, created, and curated first.

In this work, we obtain a broad, high-quality dataset of atom mapped and balanced enzymatic reactions including stereoinformation by developing a large toolbox of automated reaction curation and correction steps, which we apply to BRENDA entries on natural and non-natural substrate–product pairs. The obtained reactions comprise, to the best of our knowledge, the currently largest atom mapped database of biocatalytic transformations. The newly introduced Python code underlying this study furthermore bridges several current gaps concerning the handling of reactions, and tracking of atoms through reactions which current packages such as RDChiral^52^ or RDKit^53^ do not address yet. We then showcase how the size and quality of the obtained database, EnzymeMap, substantially improves deep learning models of enzymatic reactivity for a broad range of tasks and model architectures, leading to previously unseen accuracies, as well as performances on par with state-of-the-art organic synthesis planning tools. We make the full database and processing steps freely available.

## 2 Methods

In the following, we describe the construction of a validated, atom mapped database of balanced enzymatic reactions from BRENDA entries. We then describe the details of the models trained for retrosynthesis, forward predictions, and regioselectivity prediction. The code to reproduce all processing steps from a raw BRENDA entry to a validated, mapped reaction is available as easy-to-use Python package at https://github.com/hesther/enzymemap, along with the full dataset *via* Zenodo at https://zenodo.org/records/8254726 (raw unmapped and processed mapped reactions). The EnzymeMap Python package amounts to nearly 4000 lines of code introducing currently missing functionality for mapping reactions *via* reaction rules, correcting wrong reactions, and standardizing atom mappings, amongst many other functions. We note that for some reactions or enzyme classes BRENDA includes additional (uncurated) information not included in EnzymeMap. If one is searching for more information on a particular reaction or enzyme class such as reaction conditions, we suggest the reader check the corresponding BRENDA entry and the original literature sources.

### 2.1 Data preparation

**Fig. 1:**
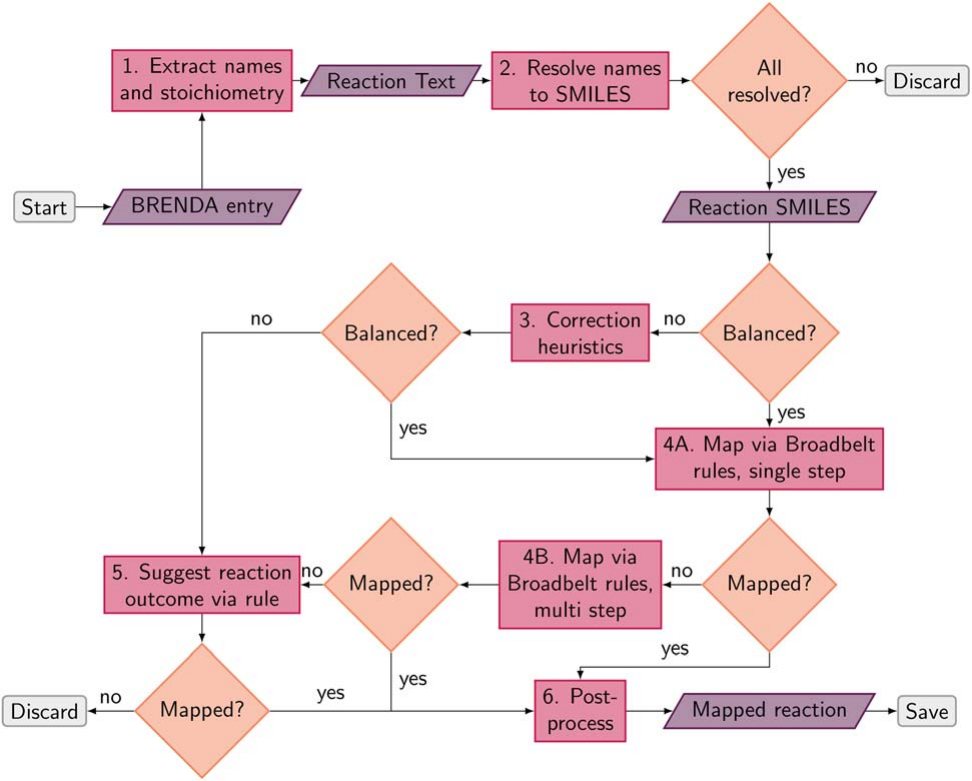
Schematic data processing pipeline to arrive at atom mapped, balanced reaction SMILES from raw BRENDA entries. Grey boxes represent the start and end points, purple parallelograms indicate input or output, pink boxes represent processes, and orange diamonds pose decision points in the pipeline.

An overview of the data processing pipeline developed in this work is given in Fig. 1. The numbering of the individual steps corresponds to the subsection numbering in the following. In general, we denote molecules (substrates, products, cofactors) by SMILES strings,^54^ and reactions by pairs of SMILES strings separated by “≫”. Enzymes were represented *via* their Enzyme Commission number (EC numbers/classes), as well as (if available) organism information and protein identifiers. The EC number is a numerical classification scheme which groups enzymatic reactions and does not specify the specific enzyme, its sequence or origin organism. For a given reaction we recommend to query an enzyme database, or tools like BridgIt^46^ to identify genes for enzymatic reactions if this information is not available.

#### 2.1.1 Processing of the BRENDA text file

**Fig. 2:**
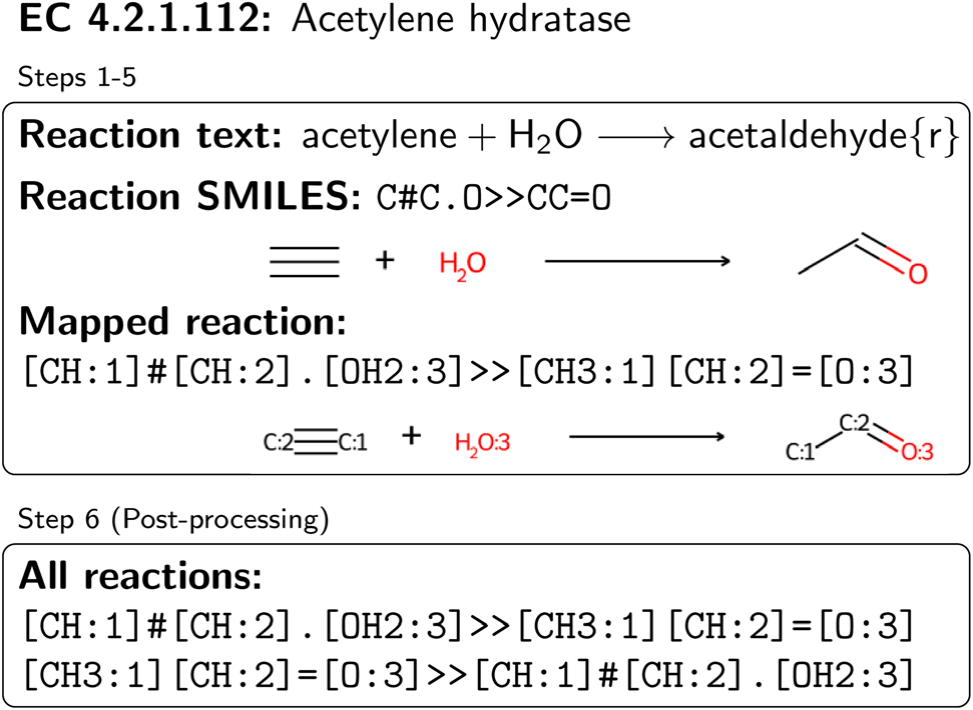
Processing of an exemplary reaction text from one substrate–product pair of acetylene hydratase. Since the reaction is tagged as reversible, two mapped and validated reactions are obtained.

BRENDA 2023-1 was downloaded from the internet (free of charge).^55^ Scripts to load BRENDA entries were taken from ref. 49, and several formatting fixes, such as missing whitespaces or hyphens were added. Raw BRENDA reactions are given as text strings, with substrates and products specified by trivial names, as well as a tag to indicate reversibility. An example entry is shown in Fig. 2, where after step 1 (extracting names and stoichiometry), the reaction text of the reservible reaction 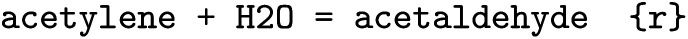 was obtained, where one molecule of acetyle and water react to one molecule of acetaldehyde. When querying BRENDA, we find that this reaction is linked to acetylene hydratases in 12 different organisms (where for nine of them reversibility is indicated), 14 literature references and one uniprot entry.

#### 2.1.2 Resolving and standardizing molecules

Next, all trivial names present in the database were attempted to be resolved to valid SMILES strings. In Fig. 2, this amounts to the names 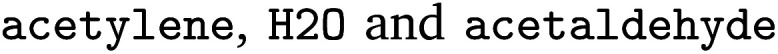. We followed six different resolving strategies, where we queried a BRENDA ligands download file whether the name is associated with an InChi or CHEBI key, and then resolved *via* InChi,^56^ CHEBI,^57^ and the trivial name. InChi keys were directly turned into SMILES strings *via* RDKit,^53^ as well as sent as queries to Pubchem.^58^ CHEBI keys were resolved *via* Pubchem either *via* a direct name query, or a synonym query. Trivial names were resolved *via* Pubchem and Opsin.^59^ All returned results were standardized, and canonicalized using RDKit, and all unique entries kept. Therefore, for a single name, multiple SMILES strings may arise. We also tested tautomerization in RDKit but found several reactions where tautomerization was not helpful, so chose to remove it. In Fig. 2 the SMILES strings 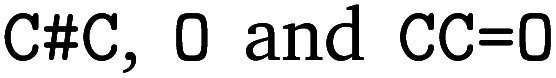 were obtained for 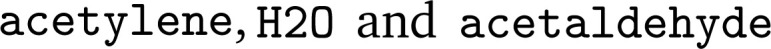, respectively, where all names were resolved to a single SMILES string. The individual SMILES strings were combined to yield reaction SMILES, where all possible combinations were taken into account if one or more names were associated with several SMILES strings. In Fig. 2 only a single reaction was obtained, namely 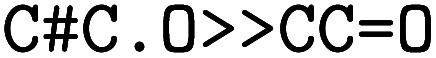. We note that step 2 can yield multiple SMILES string for a molecule name if records differ in the queried databases. In that case, all options were further considered, leading to multiple reaction SMILES for a single entry. Multiple entries were pruned later after atom mapped SMILES strings were obtained, *i.e.* in step 6.

#### 2.1.3 Correction heuristics

Each reaction was checked for stoichiometry, as well as for common mistakes concerning missing or wrong cofactors, hydrogens, or hydrogenperoxide. Detected errors were corrected in an automated fashion, such as increasing the amount of already present molecules, or adding hydrogens. Furthermore, molecules occurring in racemic mixtures were combined into a single molecule. In Fig. 2, the reaction passed all tests, and was kept as is.

#### 2.1.4 Atom mapping reactions

All balanced reactions were then atom mapped by applying the publicly available reaction rules from ref. 60 (termed “Broadbelt rule set” in the following) and tracking each atom throughout rule application. Since the Broadbelt rule set does not contain rules for reactions only affecting stereochemistry (*e.g. cis*/*trans* isomerases or racemases), we mapped reactions with the same achiral reactants and products directly without rule application. All other reactions were mapped through rule application. We first aimed to reproduce the recorded product *via* a single application of each rule. If multiple rules produced the correct product, leading to different atom mappings, only the rule changing the fewest number of atoms and bonds, as well as being most frequently applicable was used. To speed up rule application, we ordered the rules from ref. 60 based on their frequency of applicability to all BRENDA entries. In Fig. 2, this procedure yielded the mapped reaction 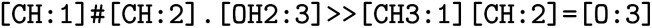. The mapped reactions were saved along with the rules that were used to produce them.

**Fig. 3:**
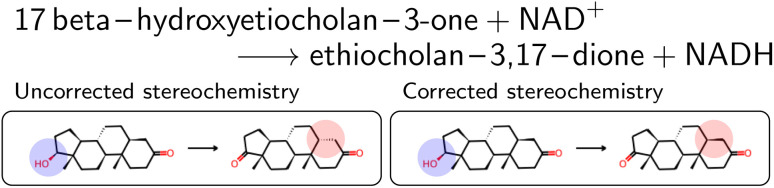
Oxidation of 17β-hydroxyetiocholan as an example of a reaction where wrong stereoisomers were retrieved. For readability, atom maps were omitted, as well as NAD+ and NADH. The reactive center is marked in blue in the reactant molecules, and the corresponding wrong chiral center in the product is marked in red.

The mapped reactions were then corrected for stereochemistry if a chiral center not participating in the reaction changed. This is usually the case when an isomeric SMILES was retrieved only for either the reactant or product, if the correct information was missing in the trivial name, or for wrong entries in the molecular databases used for name retrieval. Fig. 3 depicts an example where the wrong stereoisomer was retrieved for the product, and thus corrected. In principle, we do not know whether the stereoinformation is correct in the reactant or product. We choose to keep the stereoisomer of the reactant (concerning the chiral center which is not in the reaction center), since many product entries in BRENDA are erroneous. We note that this offers no guarantee to obtain a correct reaction, but was beneficial in a large number of cases upon manual examination.

We furthermore validated each mapped reaction, *e.g.* checked whether the products can be recreated by extracting an RDChiral^52^ template and applying it to the reactants. During the course of the project, we corrected some errors in RDChiral, available at https://github.com/hesther/rdchiral. If the template could not recreate the reaction, the mapping was dropped.

**Fig. 4:**
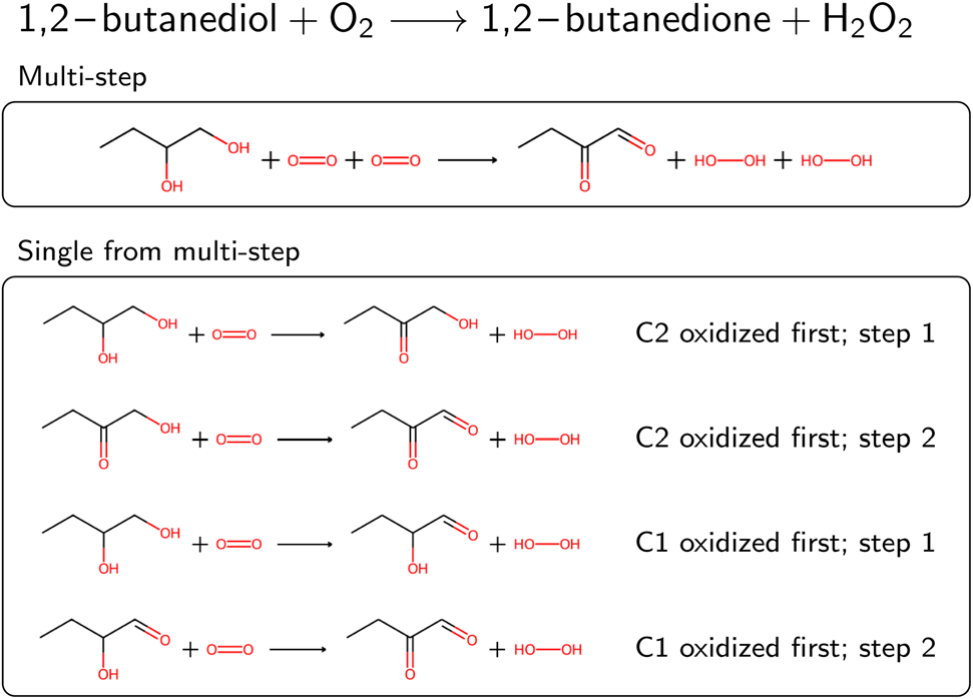
Oxidation of 1,2-butanediol as an example of a multi-step reaction requiring rule application at two sites. For readability, atom maps were omitted. The stoichiometry of the reaction was corrected (note the missing “2” in the original reaction text). The multi-step reaction can be split into four distinct single-step reactions, where either the C1 or C2 can be oxidized first.

Several reactions in BRENDA could not be mapped by a single rule application. To address this, we developed an algorithm to allow for reactions occurring at multiple sites. For example, if a reaction yields the oxidation of two hydroxyl moieties in a molecule, the respective oxidation rule needs to be applied twice. We exhaustively enumerated the outcomes of applying a rule up to two times (the maximum number of steps can be customized in our software package). To speed up this enumeration, we used only the reaction rules already present in the EC class from step 4A (or the full set if no reaction rules were recorded yet). If multiple rule applications led to the desired outcome, the overall reaction was saved, as well as all the individual steps. As an example, Fig. 4 depicts the oxidation of 1,2-butanediol, which requires rule application (oxidation by molecular oxygen) at two different sites. Since the order of the individual steps is not known, all four individual reaction steps are included in the database, and flagged with a keyword to indicate the reaction was obtained from a multi-step reaction. For individual reactions, molecules not participating were omitted, here for example the second oxygen molecule at step 1. If the obtained single-step reactions were the same due to symmetry, duplicates were dropped. Multi-step reactions as well as their corresponding single-step components were then corrected for stereoinformation similar to single step reactions.

For several reasons we chose to map reactions by applying known rules instead of mapping *via* conventional atom mapping tools.^61–63^ First of all, the available tools feature an imperfect accuracy especially for biochemical transformations.^62^ Second, recording the reaction rule used to map a reaction allows for further processing steps, such as judging the quality of a mapping, as well as the quality of the reaction itself by comparing reaction rule counts across enzyme classes. We can furthermore correct wrong stereoinformation in the products. Finally, we can easily group reactions into classes by comparing their reaction rules. Mapping *via* known rules thus offers the possibility to construct a higher quality dataset compared to simply mapping a known dataset with conventional mapping tools. We demonstrate the benefit of our mapping scheme for subsequent machine learning tasks later in this manuscript. However, we note that the coverage of reactions that can be mapped heavily depends on the chosen rule set. To the best of our knowledge, the Broadbelt rule set from ref. 60 is the most complete and validated set currently available, but even for this set a number of missing rules was identified in the course of the present study, and will be addressed in future work.

#### 2.1.5 Proposing reactions based on reaction rules

A significant number of reactions in BRENDA are unbalanced, or could not be mapped *via* reaction rules because the entry itself was flawed. Usually, the reactant was correctly extracted from literature, but the product was not entered correctly, often because it is not explicitly mentioned in the original publication. For example, the reaction 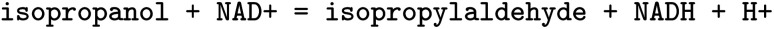, EC 1.1.1.103 wrongly lists isopropylaldehyde as product, although the oxidation of isopropanol clearly should lead to propanone. The same mistake (a ketone falsely named as aldehyde, which introduces an additional carbon atom) occurred hundreds of times in BRENDA for many different reactions, implicating a systematic error in their reaction curation. To correct for unmapped or unbalanced reactions, we propose probable reaction outcomes to correct reactions based on the reaction rules occurring in the same EC class and the similarity of both reactants and products *via* Tanimoto similarities of Morgan fingerprints^64^ as implemented in RDKit.^65^ These reactions are flagged as ‘suggested’, and may be filtered by the user. Both reactant and product similarities were taken into account to identify similar reactions based on the reactants, and then choose the most probable product after rule application, which often leads to multiple possible products due to the generic nature of the employed rule set.

#### 2.1.6 Post-processing

**Fig. 5:**
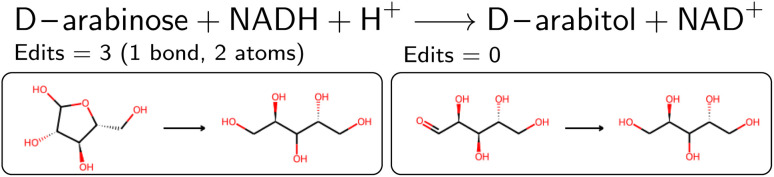
Reduction of d-arabinose. The left reaction corresponds to a combination of an closed and open form, thus wrongly assuming that the reduction includes a ring-opening reaction. We therefore choose the reaction with less edits (right panel).

As described above, a single BRENDA entry can lead to multiple valid mapped reactions if the trivial names were resolved to different SMILES strings. Although formally valid, not all reactions may necessarily be meaningful. For example, sugars were sometimes retrieved in their closed or opened form for the reactants and products respectively, as depicted in Fig. 5. We therefore counted the number of bond edits (and the number of involved atoms) and only kept the set of reactions with the minimal number of bond edits observed.

**Fig. 6:**
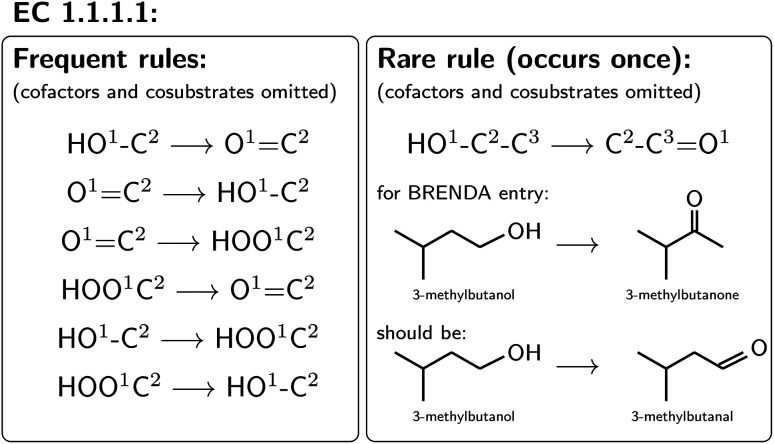
Quality of reaction based on rule count.

For all mapped reactions in an EC class, we then computed the frequency of appearance of its corresponding reaction rule, and saved its relative frequency to the column ‘quality’. This column can be used to filter the database for high-quality reactions. Often reactions with a low quality correspond to reactions that were balanced and atom mapped, but nevertheless erroneous. For example, within EC 1.1.1.1, the oxidation and reduction of hydroxy, oxy and carboxy groups occurred frequently, as depicted in Fig. 6, whereas a rule showing an oxidation plus methyl shift only occurred once, suggesting that the entry in BRENDA was possibly wrong. In fact, the depicted reaction was extracted from ref. 66 which states that the reactant 3-methylbutanol was oxidized to 3-methylbutanal (not 3-methylbutanone as listed in BRENDA). The entry thus corresponds to a wrong extraction of products in BRENDA, which we found to occur frequently.

We then standardized the obtained mappings so that the mapped reaction SMILES can be used directly to detect duplicates.

As a final post-processing step, we added the backward reactions for reversible reactions. BRENDA labels some reactions as reversible, some as irreversible and some as unknown. For reversible reaction, we directly added the backward reaction to our dataset and labeled it ‘reversed’. For reactions with unknown reversibility, we queried whether reactions with the reverse reaction template existed in the same EC class. If so, the reverse reaction was added to the dataset and labeled ‘reversed_suggested’. Altogether, we thus arrive at approximately 63k reactions (235k if protein + organism information is taken into account), yielding the largest atom mapped database of enzymatic reactions to date. If the EC class is disregarded, this boils down to 48k non-duplicate reactions.

The keywords for reversible and presumably reversible are listed in Table 1, together with all keywords for single and multi-step reactions, as well as suggested reactions based on the recorded reactants.

**Table 1:** Meaning of the columns ‘steps’ and ‘source’ in the EnzymeMap database

Flag	Description
Steps = single	Mapping *via* a single rule application
Steps = multi	Mapping *via* two rule applications (multi-step reaction)
Steps = single from multi	Single-step extracted from a multi-step reaction
Source = direct	Extracted directly from a single or multi-step BRENDA entry
Source = direct reversed	Reverse reaction was extracted directly from a BRENDA entry, which was specified as reversible
Source = direct reversed_suggested	Reverse reaction was extracted directly from a BRENDA entry, but did not include a reversibility tag
	Likely reversible based on other reactions in EC class
Source = suggested	BRENDA entry could not be mapped or was unbalanced. Product was inferred based on rule frequency and product similarity
Source = suggested reversed	Reverse of a suggested reaction where the reversibility tag indicated a reversible reaction
Source = suggested reversed_suggested	Reverse of a suggested reaction where the reversibility tag was not specified. Likely reversible based on other reactions in EC class

### 2.2 EnzymeMap database

The full EnzymeMap database is freely available *via* Zenodo at https://zenodo.org/records/8254726 as a CSV file, with columns as displayed in Table 2. The benchmarks described in this study use version 2.0 of EnzymeMap. The columns 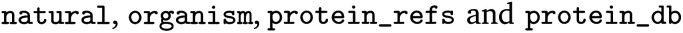 contain information as obtained from BRENDA without any curation or verification. We note that many reactions occur both as natural and non-natural reactions, often in different organisms, but sometimes also in the same organism. Since this information together with the original text of the reaction can be used to link an entry back to BRENDA, we chose to keep this information. Depending on the intended usage of the database, we recommend to first filter for the reactions of interest, and then remove duplicates stemming from information not needed for a specific application.

**Table 2:** Format of the EnzymeMap database. All entries correspond to reactions that were balanced and mapped

Column	Description
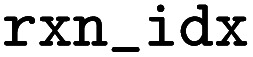	Index of the reaction in the raw file before mapping
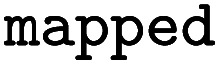	Atom mapped reaction SMILES
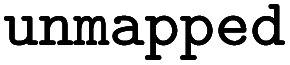	Reaction SMILES where atom maps were removed
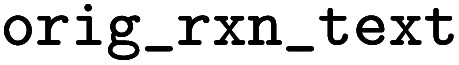	Original reaction text from BRENDA. This can be used to trace back to the original BRENDA entry, which does not have any identifier unfortunately
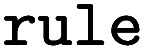	SMARTS of Broadbelt rule used for mapping
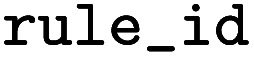	ID of the rule as distributed together with EnzymeMap
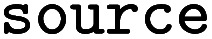	Whether entry was obtained directly, *via* reversal or *via* suggesting products, see Table 1
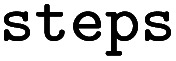	Whether entry was obtained from single or multiple rule applications, see Table 1
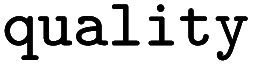	Relative frequency of rule
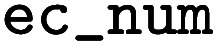	EC number
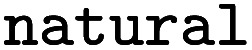	Whether reaction was classified as naturally occurring
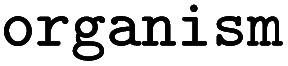	Source organism
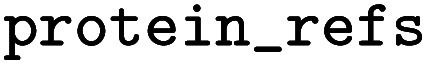	One or multiple IDs of protein sequences
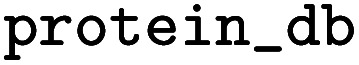	Name of database for IDs in the protein_refs column

### 2.3 Other datasets

In addition to BRENDA, this work also utilized reactions from KEGG and MetaCyc to compare the coverage and quality of atom mappings of the EnzymeMap database.

A list of KEGG enzymatic reactions given as trivial names and their EC numbers was downloaded from https://www.genome.jp/kegg-bin/get_htext (accessed 2023-07-24) amounting to 20k reactions. The reactions were then passed through the EnzymeMap workflow, leading to 10k entries where a full 4-digit EC number was specified and all names could be resolved (after step 2). 7.1k entries had at least one possible balanced reaction SMILES. After step 6, 8.0k reactions (including reversed and suggested reactions, thus more than the initial 7.1k mapped reactions) were obtained, out of which 6.6k reactions were unique disregarding the EC number.

MetaCyc version 27.0 was obtained from http://www.biocyc.org/, Copyright SRI International 2022. The 18k atom mapped SMILES strings distributed with MetaCyc did not include cofactors and were partially not balanced, so that we cross-linked the distributed atom mapped SMILES with the unmapped MetaCyc reactions to add missing molecules and cofactors. Only entries with a full 4-digit EC class and not leading to an error upon loading into RDKit were retained, amounting to 7.0k reactions. The resulting reactions (balanced SMILES strings) were then passed to the EnzymeMap workflow skipping steps 1–3. After step 6, 4.6k reactions (including reversed and suggested reactions) were obtained, out of which 4.5k reactions were unique disregarding the EC number.

### 2.4 Machine learning models

In the following, we describe the methodology and details of the employed machine learning models for retrosynthesis, forward prediction and regioselectivity prediction, which we train on the EnzymeMap database. To this end, we have selected tasks that depend on atom mapped reactions (as opposed to tasks and models taking only substrate information into account). For each task, an open-source model architecture representing the current state-of-the-art was chosen, namely (1) the single-step retrosynthesis model from ref. 31 which underlies the popular open-source synthesis planning tool ASKCOS,^67^ (2) the transformer-based single-step forward and retrosynthesis model of the successful IBM RXN model,^68–71^ and (3) the message-passing neural network Chemprop which recently was shown to produce high-quality reaction predictions.^72–74^

#### 2.4.1 Template-based retrosynthesis *via* neural networks

To assess the improvements EnzymeMap offers over other databases, we train a neural network model to rank relevant retrosynthetic templates to produce a given product, as also used in the open-source synthesis planning tool ASKCOS.^67^ The model therefore suggests reaction templates given an input molecule, where we use RDChiral^52^ to produce chiral reaction templates and retrain the template-relevance model from ref. 31 with the default hyperparameters described therein. Since the model is trained as classification task (*i.e.* to identify the template that led to the given product), it is important that all templates extracted from a dataset are mutually exclusive. We thus used the code from ref. 75 to arrive at exclusivity-corrected templates.

The model itself takes product Morgan fingerprints of length 2048 and radius 2 as input, and uses a single hidden layer of 2048 neurons with RELU activation functions to map the fingerprint to its retrosynthetic template. A dropout rate of 0.2 was applied. The learning rate was set to 0.001, with early stopping if the validation error did not improve for three consecutive epochs.

Three different datasets were employed. RHEA,^50^ exactly as provided in ref. 44, as well as MetAMDB^76^ (which is based on BKMS-react^77^) and EnzymeMap (this work, based on BRENDA^51^), since these datasets contain balanced atom mapped reactions. MetAMDB consists of approximately 43 000 reactions with atom mappings generated by the Reaction Decoder Tool,^61^ and thus constitutes the largest atom mapped reaction set prior to the current study, serving as a comparison to EnzymeMap regarding quantity. RHEA was chosen because it is highly curated and reliably, thus serving as a comparison regarding quality. Furthermore, it was utilized in a recent bioretrosynthesis study.^44^ For MetAMDB and EnzymeMap, we follow the RHEA preparation instructions from ref. 44 closely. Namely, we removed all products occurring more than 100 times in the dataset to get rid of common cofactors or other frequent molecules such as protons, and then deleted molecules from the reactants that had no matching atoms in the products. We then kept all reactions that had a single product molecule. Importantly, duplicates were removed for all reactions, which might occur where *e.g.* reactions differed only in their cofactors before cofactor removal. This creation of single-product reactions is necessary for the chosen model architecture, which can only take a single product as input. For EnzymeMap, this led to 18k reactions and 2.8k templates. For MetAMDB, 14k reactions and 5k templates were obtained. For RHEA, 8k reactions and 4k templates were obtained. All datasets were randomly split to 80% training, 10% validation and 10% test sets. We used the entire dataset, including templates that occurred infrequently or even only once. This made our modelling task much more challenging compared to approaches that exclude templates with few reaction precedents. We furthermore report ablation studies with the same test set as EnzymeMap for MetAMDB, RHEA, and subsets of EnzymeMap, were all identical reactions to this common test set were removed and the remainder of reactions split into 80% training and 20% validation sets, respectively. In another ablation study, we remapped EnzymeMap (either raw from BRENDA, or after the EnzymeMap workflow) using the popular transformer-based atom mapping software RXNMapper,^63^ which is the current state-of-the-art mapper regarding versatility and accuracy. We used default settings as provided in the RXNMapper Python package.^78^

We report top-*N* accuracies for all models, *i.e.* the ratio of test datapoints where the correct template was identified in the top-*N* suggestions. Although this metric is associated with a few shortcomings concerning its translation to real-world performance for retrosynthesis tasks,^79^ it allows us to compare our approach to performances for organic retrosynthesis, which is typically reported using top-*N* accuracies.

#### 2.4.2 Forward reaction and retrosynthetic pathway prediction models based a transformer model

To assess the importance of a large and diverse dataset for deep learning reaction models, we retrained a recently published enzymatic reaction prediction tool^43^ within the IBM RXN toolbox,^68–71^ based on a transformer architecture, and originally trained on the ECREACT dataset.^43^ Both the forward-reaction prediction, as well as the retrosynthesis prediction were retrained exactly as described in ref. 43 after adding the respective reactions from EnzymeMap on top of ECREACT and deduplication. The original model was retrained, too, to ensure that the correct values were reproduced. In detail, the models ‘EC3’ were retrained, which utilize the first three digit of the EC number in the reaction SMILES, for example for the EC number 1.1.3.2:
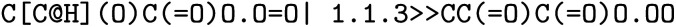


The forward model predicts the product side, 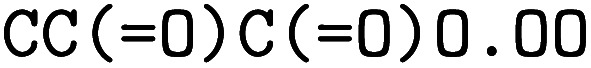 based upon the reactant side, 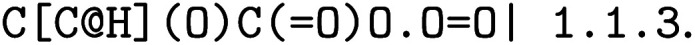. The backward model takes the product side as input and predicts the reactants, including the three-digit EC number. Each model consisted of a transformer encoder and decoder with 4 layers, a word vector and RNN size of 384, positional encoding turned on, 8 attention heads with global and self attention, adam as optimizer with *β*_1_ = 0.9 and *β*_2_ = 0.998, a learning rate of 2.0 with the noam decay method, and a dropout rate of 0.1. Batches of 6144 were used. Both models were trained on an OpenNMT^80^ Version adapted for molecules, see ref. 43 for further details.

Since the models do not require atom mapped reactions, we use the full set of reactions after step 2 in Fig. 1, amounting to 100k reactions, where only duplicates within an EC class (but not across EC classed) were removed, since the model makes use of the EC class. After tokenization at the third-digit EC class, and adding the ECREACT reactions, we arrive at 90k unique reactions (as opposed to 57k reactions with only ECREACT). In this benchmark, we do not showcase the capabilities of the EnzymeMap correction and curation pipeline (since the reactions are unmapped and uncorrected), but only the influence of the size of the dataset and the number of reactions per EC class. The 100k reactions contain also unbalanced reactions, as well as those that could not be mapped in our processing pipeline, since we expect the transformer model to still be able to make use of them. Furthermore, if different options to resolve a trivial name to a SMILES string were found, the dataset contained all options, which explains the large size of 100k *versus* 63k reactions in EnzymeMap.

To further showcase the effect of data curation, correction and validation, we retrain both the forward and backward model with ECREACT with only processed EnzymeMap reactions (*n* = 63k) added. After tokenization, the dataset amounts to 84k reactions, so less than in the previous benchmark where the raw reactions were added, but at higher quality.

We report top-*N* accuracies for the forward prediction, backward prediction, and roundtrip task (backward prediction followed by single forward prediction).

#### 2.4.3 Regioselectivity models based on graph-convolutional neural networks of the transition state

Lastly, we showcase the importance of the underlying dataset when predicting the regioselectivity of enzymatic reactions. We again use the single-product versions of RHEA, MetAMDB, and EnzymeMap described above for the template-relevance model. For EnzymeMap, only reactions obtained *via* a direct, single rule application were used. For all reactions in the respective datasets, reaction templates were extracted *via* RDChiral.^52^ The templates were then applied to the reactants, and all reactions were kept that produced more than one possibility for the products. Thus, regioselective reactions were identified, *i.e.* where multiple sites could have reacted in theory. Recorded reactions were labeled “1” and all other reactions as “0”. For example, if the application of a rule extracted from 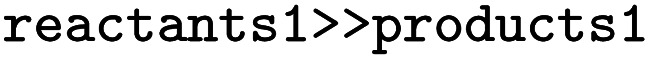, produced 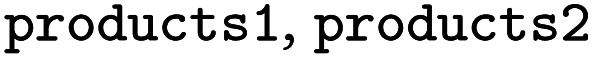 and 
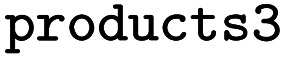
 the following three lines were added to the dataset:
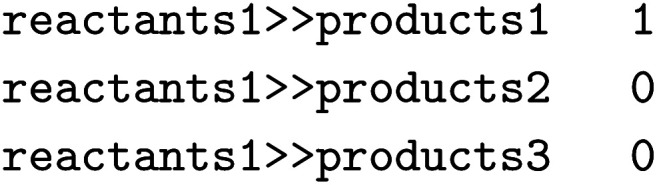
where 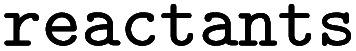 and 
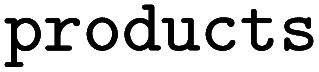
 are SMILES strings. For EnzymeMap, this led to 21k reactions from 5.1k reactants. For MetAMDB, 15k reactions from 3.3k reactants were obtained. For RHEA, 7.6k reactions from 1.9k reactants were obtained. All datasets were split randomly into 80% training, 10% validation and 10% test data.

We note that the models did not make use of the EC class or protein information, and therefore only learn which reaction outcomes are more likely in a general sense, and not specific to a protein. Reactions with different outcomes based on the EC class or protein identity thus add noise to the dataset, so that even a perfect model cannot reach 100% accuracy. We did not include the EC class or protein sequence, since recent work identified major shortcomings in current approaches to encode the protein information in a meaningful way even for highly curated data.^81^

We then trained a classification model on the full training sets, as well as random subsamples of differing sizes. We chose the graph-convolutional neural network architecture Chemprop^72^ with reaction support from ref. 73 (CGR-Chemprop) for our classification models, since this framework was recently demonstrated to learn high-accuracy reaction properties such as barrier heights, rates, and regioselectivities.^74^ CGR-Chemprop relies on transforming reaction SMILES to their corresponding condensed graph of reaction, an overlay between the reactant and product graphs, *i.e.* the artificial, graph-based transition state of the reaction. We trained for 100 epochs and used 10-fold cross-validation for each prediction task since the performance is very sensitive to the data split. All other hyperparameters such as number and size of layers, or learning rates were kept at their default values (three rounds of message passing with hidden size of 300, mean aggregation over the graph, 2 feed-forward layers with a hiden size of 300). We report the flat accuracy of the classification, *i.e.* the model's ability to discern between reactive and non-reactive data points. For example, if the classification model predicted 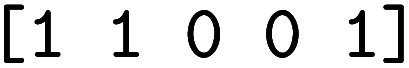 for the test set
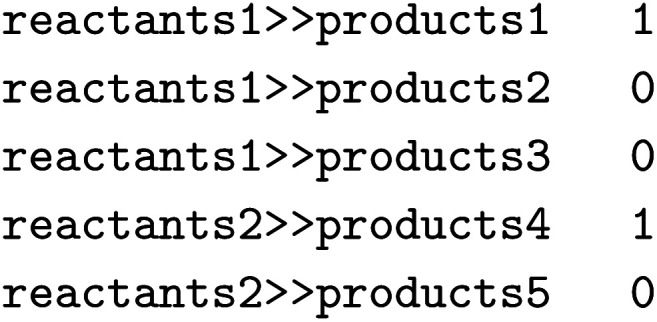
the accuracy (percentage of correct predictions) would be two out of five, *i.e.* 20%. We furthermore report the top-1 accuracy to identify the correct products given the reactants, *i.e.* the fraction of test data points where the reaction labeled “1” had a higher raw predicted value than all reactions labeled “0” originating from the same reactants and template. For the example above, if the raw scores from the model (prior to applying a threshold to create binary labels from the continuous predictions) were 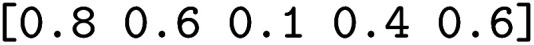, then the model scored the correct products at rank 1 for 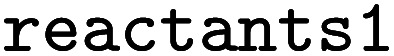, and at rank 2 for 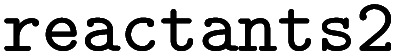, leading to a top-1-accuracy of 50%.

## 3 Results and discussion

### 3.1 Database analysis

Table 3 lists the number of mapped reactions that were obtained *via* direct mapping of the original entry *via* a single reaction rule application (step 4A in Fig. 1), *via* multiple rule applications (step 4B in Fig. 1), as well as *via* suggesting products based on the reactants of unmapped or unbalanced reactions (step 5 in Fig. 1). In total, 63 137 reactions with a unique mapped reaction within an EC class were obtained, not taking into account information on natural/non-natural substrates, organisms and proteins. Disregarding the EC class, 47 974 unique reactions were obtained. When also discerning between natural/non-natural substrates, organisms and proteins, a total of 234 845 non-duplicate entries is obtained. The majority of the reactions in EnzymeMap (71%) stem from single step reactions from the original BRENDA entries. 17% of non-duplicate entries (21% taking into account organisms and proteins) stem from BRENDA entries classified as “natural substrate/product pair”.

**Table 3:** Number of mapped and unmapped reactions without duplicates in EC class (and with protein/organism information in third column), where the mapping could either be inferred from the originally recorded database entry *via* one or multiple steps of reaction rule application, or was suggested from reaction rules in the same EC class

	#	# with prot	# EC 1	# EC 2	# EC 3	# EC 4	# EC 5	# EC 6
Mapped from original, single step	44 894	162 322	10 621	12 263	16 622	2698	1414	1266
Mapped from original, multi-step	1122	3195	428	134	444	64	46	6
Single-step reactions *via* splitting multi-step	2269	5811	550	319	1270	61	47	22
Mapped from suggestion, single step	6327	13 133	1425	1797	2514	365	96	130
Reversed reactions known reversibility	6929	46 230	2899	2301	364	539	740	86
Reversed reactions suggested reversibility	6681	28 897	3813	320	916	1350	84	198
Mapped with natural substrate/product	10 558	48 570	3791	2952	2103	907	565	240
Unbalanced	8599	17 307	3121	1805	2638	717	139	179
Balanced but unmapped	5841	20 005	2866	1074	1011	585	160	145

**Fig. 7:**
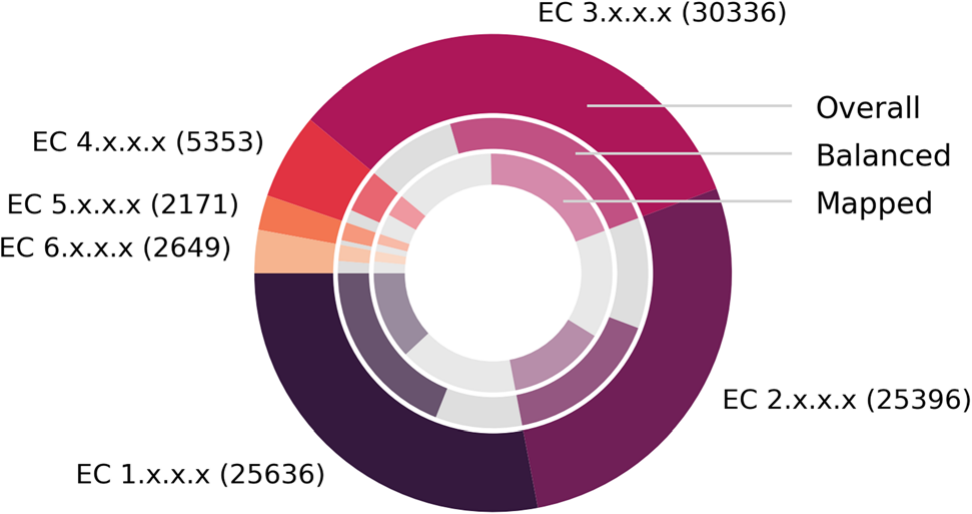
Composition of BRENDA (without natural/non-natural, organism and protein information). (Outer ring): number of reactions per EC class. (Middle ring): fraction of balanced (colored) and unbalanced (gray) reactions. (Inner ring): fraction of reactions that were atom mapped (colored, multiple mappings per BRENDA entry were only counted once) or remain unmapped (gray). Since unbalanced reactions can be mapped *via* step 5 of our workflow, this number might be larger than the number of balanced reactions. Exact numbers in Table 3.

Fig. 7 depicts the number of mapped reactions per EC class, split into overall (outer circle), balanced (second circle) and mapped (inner circle) reactions, after all pre- and post-processing steps. We find that no EC class exhibits a disproportionally large number of imbalanced reactions or failed mappings, and that EnzymeMap is mainly comprised of reactions in the EC classes 1 (oxidoreductases), 2 (transferases) and 3 (hydrolases) to nearly equal amounts, whereas the EC classes 4 (lyases), 5 (isomerases) and 6 (ligases) only make up about 10% of all reactions in total. Translocases (EC 7) were not considered for mapping, since they catalyze the movement of ions or molecules across membranes, so that the reactant and product molecules are usually the same.

**Fig. 8:**
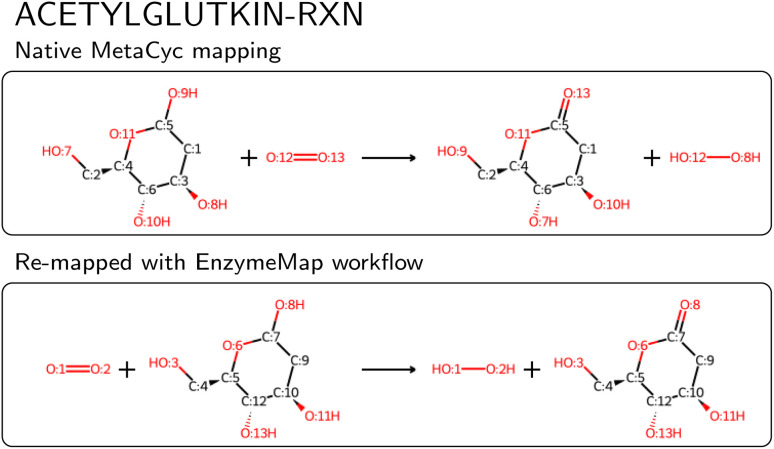
Example of a wrong atom map in MetaCyc for the entry ‘ACETYLGLUTKIN-RXN’.

We then compared the obtained reactions to KEGG and MetaCyc after removing atom mappings, and found that of the nearly 48k unique reactions in EnzymeMap, only 0.5k are also found in MetaCyc (0.6k after passing MetaCyc through the EnzymeMap workflow, *i.e.* also adding reverse reactions), and 1.8k are also found in KEGG (5k after passing KEGG through the EnzymeMap workflow), where we compared the unmapped reaction SMILES strings. EnzymeMap thus provides access to several tens of thousands of reactions not covered by other databases. The workflow furthermore is not limited to BRENDA. With minimal changes to the setup of the initial raw reactions, we were easily able to map reactions from KEGG and MetaCyc, leading to 8.0k and 4.6k mapped reactions, respectively. We distribute those alongside EnzymeMap, since a combination of all sources might be beneficial for future data-driven prediction of enzymatic reactions. For MetaCyc, we were furthermore able to compare the atom mappings distributed with MetaCyc with atom mappings from the EnzymeMap workflow. For reactions that led to a valid mapped reaction with our workflow, we found same mappings for 70% of the reactions (accounting for equivalent mappings due to symmetry). For the reactions with different atom mappings, our mappings had an equal or less number of bond edits than the MetaCyc mappings in 92% of cases. Manual examination of random cases where EnzymeMap lead to better mapping results than the native MetaCyc mappings revealed a few hundred of errors in MetaCyc, with an example shown in Fig. 8, where five atoms are wrongly mapped. We also found cases where atoms corresponding to different elements (for example phosphorus and carbon) were assigned the same map number. This highlights the need for better atom mapping routines for enzymatic reactions, or at least better post-processing steps to flag erroneous reactions, even for highly curated databases like MetaCyc. However, the EnzymeMap workflow is not without flaws itself, due to missing rules in the employed Broadbelt rule set, leading to possibly wrong mappings or no mappings at all. For the latter case, we compared the number of MetaCyc reactions that could not be mapped *via* the EnzymeMap workflow per 1-digit EC class, and found that all EC classes are about equally affected by failed mapping attempts. Manual inspection revealed many reactions in MetaCyc, for which no rule exists currently, including multi-step reactions involving different reaction steps (*e.g.* a reaction followed by decarboxylation), reactions involving changes in rings, some transfer reactions, ring-forming reactions, and hydrolysis of peptide bonds, amongst others. We attempt to address missing rules in a future publication, and thus future versions of EnzymeMap. Since a new rule set only requires to update one file and re-run the workflow, the incorporation of new rules is a trivial exercise. For the remainder of this study (and the current version of EnzymeMap), we stick with the Broadbelt rule set, since the creation and validation of a new rule set is well beyond the scope of the current study.

### 3.2 Retrosynthesis based on neural-network

**Fig. 9:**
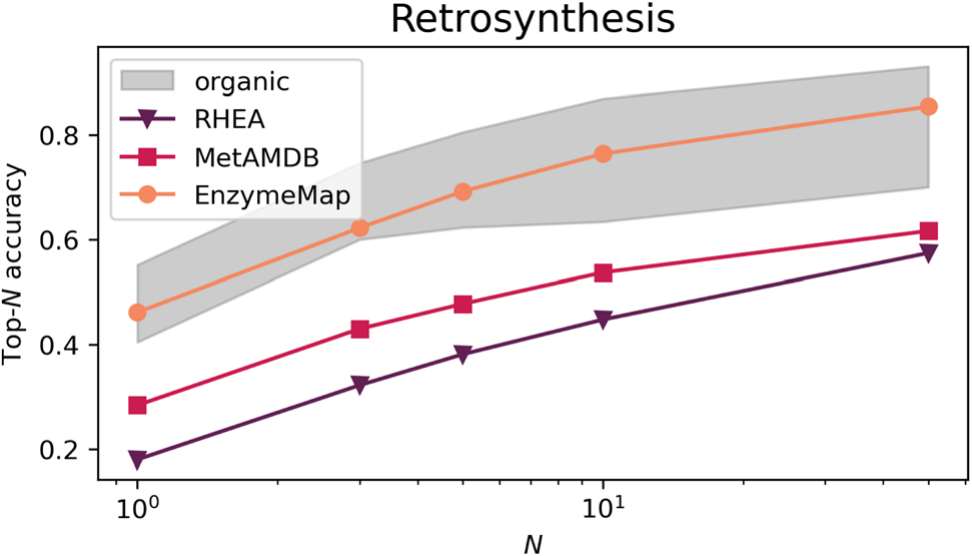
Top-*N* accuracies for retrosynthesis models trained on different databases (RHEA, MetAMDB and EnzymeMap). The gray area corresponds to top-*N* accuracies typically achieved by organic retrosynthesis models on the USPTO-50k dataset.

Fig. 9 depicts the top-*N* accuracy of neural networks trained on identifying templates which lead to the recorded precursors, *i.e.* predicting retrosynthetic single-step pathways. The gray area corresponds to top-*N* accuracies typically achieved by organic retrosynthesis models for the USPTO-50k dataset, with values taken from ref. 82, 83 and 75 including neural network models on fingerprints, graph-convolutional neural networks on graphs, and transformer models on strings or graphs. Although these are different models trained on different datasets (*i.e.* not directly comparable), they showcase an important prerequisite for computer-aided retrosynthesis, namely that powerful one-step synthesis models are needed, which can subsequently be used in multi-step synthesis planning tools. Using EnzymeMap, for the first time, an enzymatic retrosynthesis model is able to compete with the accuracy of organic retrosynthesis tools, which is remarkable given that the model is very simple, and was not optimized in any way. In comparison, models trained on MetAMDB or RHEA feature a relatively low accuracy. A low top-*N* accuracy is especially problematic for designing multi-step synthesis pathways, where clever ranking algorithms are essential to navigate the combinatorially explosive number of template application possibilities at each reaction step. For instance, using the low top-1-accuracy of 0.18 for RHEA from Table 4, designing a pathway of three independent steps by taking the top-1 template at each step, only 0.18^3^ = 0.006 = 0.6% of test products would yield the correct synthesis pathway. In practice, this would be even lower since we do not want to find just any pathway consisting of three reactions that produce the product, but pathways starting from *e.g.* buyable materials, or having no unnecessary loops of protection and de-protection. In comparison, 9.7% of test products (0.46^3^ = 0.097) would be recovered using the EnzymeMap model (taking only the top-1 recommendation), which is an about twenty-fold increase in success rate. We therefore anticipate EnzymeMap to also perform well with multi-step retrosynthesis models, although the training of such models *via e.g.* Monte Carlo Tree Search or Reinforcement Learning is beyond the scope of the current study.

**Table 4:** Top-*N* accuracies for retrosynthesis models trained on different databases (RHEA, MetAMDB and EnzymeMap)

*N*	1	3	5	10	50
**Internal test set**					
RHEA	0.18	0.32	0.38	0.45	0.57
MetAMDB	0.28	0.43	0.48	0.54	0.62
**EnzymeMap**	**0.46**	**0.62**	**0.69**	**0.76**	**0.85**
raw BRENDA + RXNMappper	0.32	0.44	0.51	0.57	0.67
EnzymeMap + RXNMapper	0.35	0.51	0.58	0.65	0.75

**Same test set**					
RHEA	0.04	0.08	0.09	0.10	0.15
MetAMDB	0.23	0.35	0.40	0.45	0.56
EnzymeMap					
• No multi, no rev., no sugg	0.35	0.51	0.57	0.64	0.72
• No reversed, no suggested	0.38	0.54	0.59	0.66	0.75
• No suggested	0.39	0.55	0.62	0.69	0.78
• No reversed	0.40	0.56	0.63	0.70	0.79
• No multi	0.43	0.61	0.68	0.76	0.83
• **All**	**0.46**	**0.62**	**0.69**	**0.76**	**0.85**

Table 4 furthermore lists several ablation studies to showcase the importance of the data curation, cleaning, and validation routines developed in this study. First, we explore whether mapping the resolved raw reaction SMILES from BRENDA *via* an alternative route to Fig. 1 affects the performance of retrosynthesis models. To this end, we map the first reaction SMILES per BRENDA entry using the state-of-the-art transformer-based atom mapper RXNMapper.^63^ This leads to a large loss in performance yielding top-*N* accuracies close to those achieved by the MetAMDB model. We therefore conclude that the careful mapping, correction, and validation approach for enzymatic reactions developed in this work is essential for a good performance of reaction prediction models. Second, we take the cleaned and curated reactions from EnzymeMap and re-map them with RXNMapper. This also leads to a large loss in performance, although to a less extent, resulting in top-*N* accuracies below the performances typically achieved for organic reactions. We therefore conclude that also the quality of atom maps is essential for training reaction prediction models, and that simple, uncurated mapping impacts the performance negatively. This ablation study therefore showcases the importance of database quality.

We then retrain all models and test them on the EnzymeMap test set, after removing overlap in the training and validation sets with the new test set. For RHEA and MetAMDB, this provides an even harder task trying to predict retrosynthesis steps of reactions for which no close analogue might be available in the training set, so that the low performances in Table 4 were somewhat expected. The performance loss is less for MetAMDB *vs.* the much smaller RHEA, indicating that MetAMDB covers a wider range of reaction functionalities.

We then removed reactions from the EnzymeMap training and validation sets in a further set of ablation studies to benchmark the importance of database quantity. The performance degrades for every loss of information, starting with omitting multi-step reactions, reverse reactions, suggested reactions, and combinations thereof, showcasing the value and need for each of the processing steps in the EnzymeMap workflow. The increased coverage of enzymatic reaction space by adding multi-step reactions, reverse reactions, or correcting entries *via* suggesting outcomes (instead of dropping the entry) is beneficial to the performance of the trained retrosynthesis model, highlighting the need for larger reaction databases.

The ablation studies therefore highlight the importance of the developed data generation/curation approach both in terms of quality and quantity of the obtained reactions. Although our approach adds computational burden compared to out-of-the-box mapping strategies, the performance improvement in machine learning models warrants its use, and, for the first time, enables accurate data-driven modeling of enzymatic synthesis planning.

### 3.3 Forward reaction prediction and retrosynthesis based on transformers

Next, we showcase the merits of EnzymeMap for large deep-learning models not dependent on atom mappings, *i.e.* benchmark only the effect of increased coverage, but not quality. The IBM RXN-for-Chemistry platform provisions forward and backward models for biocatalysed reaction predictions. Both models have been trained using a multi-task transfer learning approach on a transformer architecture.^43^ The multi-task transfer learning regime consisted of samples from two training sets: the USPTO data set containing 1 million organic reactions and the data set ECREACT consisting of 56 579 biocatalysed reactions sourced from the databases RHEA, BRENDA, PathBank, and MetaNetX.^84–87^ Including the enzyme commission numbers during training enables the backward model to not only predict the substrates based on a product, but also the class of the catalysing enzyme. While the model showed state-of-the-art performance, it was held back by the limited availability of enzyme-catalysed reactions. As ECREACT contained only 11 130 reactions extracted from BRENDA, retraining the models using the considerably larger non-atom mapped version of EnzymeMap in addition to ECREACT leads to a 59% increase in training set size (at an overall dataset size of 90 028), which results in an increase in the mean predictive accuracy of sub-classes in the forward model across all enzyme classes and an increase in the mean predictive accuracy of the retro model with the exception of transferases and ligases, see Table 5. As the new data introduced with the inclusion of EnzymeMap not only adds new samples for the regions of reaction space covered by ECREACT but also increases the covered space, the increase in dataset size does not always lead to an increase in predictive accuracy. Indeed, the addition of diverse data can even lead to a (insignificant) decrease in predictive accuracy as is the case for ligases in the backwards model.

**Table 5:** Top-*N*-accuracies of the forward, backward and roundtrip prediction task with the IBM Rxn-for-Chemistry transformer models with 3-digit EC classes trained on different datasets. Training with ECREACT reproduces the results reported in ref. 43. Additionally adding all raw, unprocessed reactions increases the performances for most EC classes and tasks. When utilizing only the validated EnzymeMap reactions, the performance further increases

		Forward				Backward				Roundtrip			
		*N* = 1	*N* = 3	*N* = 5	*N* = 10	*N* = 1	*N* = 3	*N* = 5	*N* = 10	*N* = 1	*N* = 3	*N* = 5	*N* = 10
ECREACT^43^ (*n* = 59 579)	Overall	0.49	0.59	0.64	0.69	**0.60**	**0.67**	**0.69**	**0.71**	**0.40**	**0.42**	**0.42**	**0.43**
	EC 1	0.27	0.45	0.51	0.57	0.19	0.28	0.31	0.38	0.08	0.10	0.11	0.13
	EC 2	0.64	0.69	0.73	0.78	**0.86**	**0.90**	**0.91**	**0.91**	**0.61**	**0.62**	**0.62**	**0.63**
	EC 3	0.39	0.58	0.62	0.67	0.31	0.43	0.45	0.48	0.19	0.25	0.26	0.27
	EC 4	0.28	0.38	0.41	0.44	0.46	0.62	0.64	0.67	0.18	0.20	0.20	0.21
	EC 5	0.15	0.27	0.32	0.39	0.19	0.25	0.29	0.31	0.05	0.07	0.07	0.07
	EC 6	0.34	**0.55**	**0.61**	0.63	**0.44**	**0.55**	**0.56**	**0.58**	**0.26**	**0.29**	**0.29**	**0.29**
ECREACT + raw EnzymeMap (*n* = 90 028)	Overall	0.49	0.60	0.65	0.70	0.52	0.61	0.64	0.66	0.32	0.35	0.36	0.37
	EC 1	0.32	0.47	0.54	0.60	0.22	0.31	0.36	0.40	0.09	0.13	0.15	0.16
	EC 2	0.63	0.70	0.74	0.78	0.80	0.86	0.88	0.89	0.52	0.54	0.54	0.54
	EC 3	0.45	0.60	0.65	0.70	0.30	0.41	0.46	0.48	0.24	0.29	0.31	0.32
	EC 4	0.39	0.57	0.62	0.65	0.45	0.60	0.64	0.67	0.21	0.26	0.28	0.30
	EC 5	**0.29**	**0.46**	**0.52**	**0.56**	0.23	**0.40**	**0.46**	**0.48**	**0.11**	**0.16**	**0.18**	**0.18**
	EC 6	**0.35**	0.49	0.58	0.63	0.40	0.44	0.46	0.49	0.23	0.26	0.27	0.29
ECREACT + processed EnzymeMap (*n* = 83 470)	Overall	**0.54**	**0.67**	**0.72**	**0.76**	0.55	0.64	0.67	0.69	0.37	0.41	0.42	0.43
	EC 1	**0.39**	**0.60**	**0.66**	**0.71**	**0.23**	**0.34**	**0.37**	**0.42**	**0.12**	**0.16**	**0.18**	**0.19**
	EC 2	**0.65**	**0.72**	**0.75**	**0.81**	0.81	0.87	0.88	0.89	0.58	0.60	0.61	0.61
	EC 3	**0.59**	**0.74**	**0.79**	**0.82**	**0.38**	**0.54**	**0.57**	**0.62**	**0.30**	**0.38**	**0.40**	**0.42**
	EC 4	**0.41**	**0.63**	**0.68**	**0.71**	**0.52**	**0.62**	**0.65**	**0.69**	**0.24**	**0.28**	**0.29**	**0.32**
	EC 5	0.25	0.38	0.46	0.54	**0.23**	0.38	0.42	0.46	0.08	0.14	0.14	0.15
	EC 6	0.28	0.54	0.60	**0.66**	0.29	0.33	0.37	0.37	0.14	0.17	0.17	0.17

The above benchmarks showcase the effect of a sheer increase in dataset size, but not necessarily dataset quality. We therefore also retrained all models with only the processed and validated EnzymeMap reactions + ECREACT (with an overall dataset size of 83 470). Here, we find even further improvements compared to the raw EnzymeMap dataset for nearly all enzyme classes in both the forward and reverse model. Thus, even for unmapped reactions, curating a high-quality dataset can have beneficial effects to models trained on them, highlighting the need of both high-quality and high-quantity datasets for chemical deep learning.

Overall, EnzymeMap leads to a large performance increase in both the forward and retro model. The retrained models (on the previous version 1 of EnzymeMap) have been made available as the default biocatalysis models on the IBM RXN-for-Chemistry platform (https://rxn.res.ibm.com).

### 3.4 Regioselectivity prediction based on graph-convolutional neural network

**Fig. 10:**
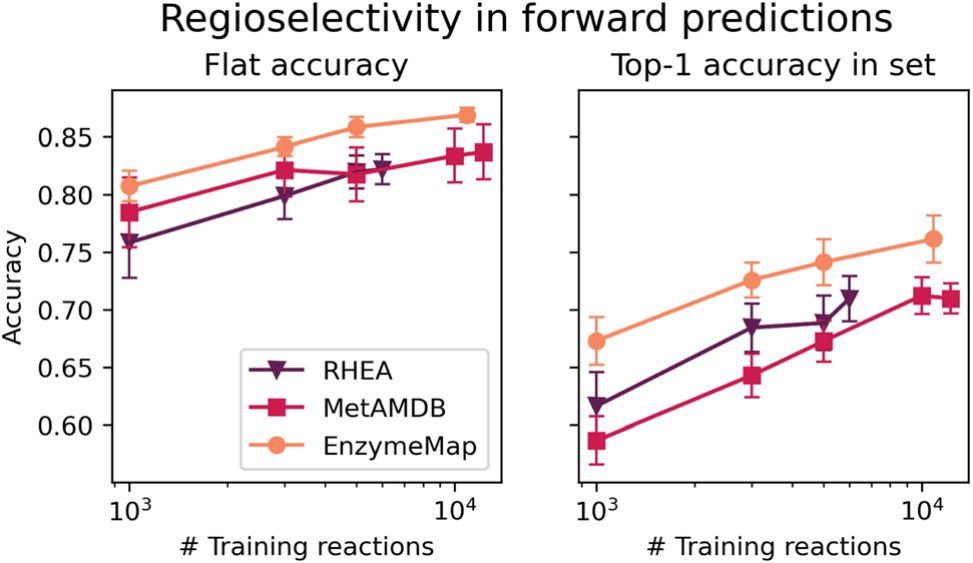
(Left) Classification accuracy of models trained on different databases (RHEA, MetAMDB and EnzymeMap) for predicting the regioselective outcome of a reactions. (Right) top-1 accuracy for identifying the most probable outcome given a set of reactants. Errorbars correspond to 95% confidence intervals from 10-fold cross-validation.

Fig. 10 depicts the classification accuracy of CGR-Chemprop trained on regioselective reactions from EnzymeMap, MetAMDB, and RHEA, as well as the fraction of data points in the test set where the true product was ranked highest (as opposed to false products obtained *via* template application to different sites in the reactants) in dependence on the number of training reactions. For the full training sets, Table 6 furthermore lists the achieved accuracy and top-1 accuracy. As evident in Table 6, EnzymeMap offers a large performance boost for regioselectivity predictions for the full datasets (right-most data points in Fig. 10), but also for random subsets of the training data. Interestingly, we find a better top-1 accuracy for RHEA *vs.* MetAMDB when trained on the same number of datapoints (right panel in Fig. 10), which underpins the quality of RHEA which is heavily curated. Similarly, we conclude that EnzymeMap not only offers a benefit in size and diversity (*i.e.* coverage of reactions), but also in its low inherent noise obtained *via* the extensive validation and curation efforts described in the Methods section. Through comparison of different training sizes, we conclude that both the quantity and quality of recorded reactions influence the performance of reaction prediction models, giving EnzymeMap a large benefit over other databases. With the full dataset utilized, we obtain a flat accuracy of 87% to discern between reactive and non-reactive reaction instances, as well as a top-1 accuracy of 76% to identify the correct regioselective reaction outcome given a set of product options. Together with the high accuracies reported for retrosynthesis and forward prediction, this lays the groundwork of successful data-driven biocatalytic synthesis design. We anticipate further performance improvement upon inclusion of enzyme information to the model input, so that the model can learn different regioselectivies exhibited by different enzymes (which in this study only manifests as aleatoric, irreproducible error).

**Table 6:** Classification accuracy and top-1 accuracy of CGR-Chemprop models trained on different databases (RHEA, MetAMDB and EnzymeMap) for predicting the regioselective outcome of reactions

	accuracy	top-1 accuracy
RHEA	0.82	0.71
MetAMDB	0.84	0.71
**EnzymeMap**	**0.87**	**0.76**

## 4 Conclusion

We have developed a database curation, validation, and mapping pipeline for enzymatic reactions, producing an extensive dataset of atom mapped, balanced, validated, and diverse reaction which include correct stereoinformation from BRENDA entries. We showcase that this new dataset, EnzymeMap, is sufficiently large for data-driven deep learning and offers significant performance improvements over all previous databases for retrosynthesis, forward prediction and regioselectivity prediction tasks. For the first time, we report prediction performances on par with organic retrosynthesis tools. This performance boost comes from both better coverage (larger number of total reactions, larger number of reactions per EC class), as well as better quality regarding the reactions itself and their atom mappings. We distribute the full pipeline as easy-to-use Python package, and demonstrated that the workflow can be easily adapted to other databases such as MetaCyc or KEGG. We expect that the EnzymeMap database will spark numerous inventions in the field of computer-aided enzymatic reaction prediction, especially for applications relying on mapped, balanced reactions such as the computer-aided design of enzymatic cascades.

## Data availability

The EnzymeMap dataset version 2.0 (along with the re-mapped KEGG and MetaCyc files) is available *via* Zenodo at https://zenodo.org/records/7841781 as a machine- and human-readable CSV file containing the EC number, the original reaction text, mapped and unmapped reaction SMILES, as well as further metadata as described in the methods section. The code to reproduce all processing steps from a raw BRENDA entry to a validated, mapped reaction is available as easy-to-use Python package at https://github.com/hesther/enzymemap. The repository furthermore contains scripts for the retrosynthesis and regioselectivity models. Instructions to retrain the transformer model are available in ref. 43 and were used as provided. All employed software packages are freely available on GitHub: https://github.com/rxn4chemistry/rxnmapper for RXNMapper, https://github.com/hesther/rdchiral for custom RDChiral, https://github.com/hesther/templatecorr for the hierarchical template correction. BRENDA is freely available online at http://brenda-enzymes.org/.

## Author contributions

E. H. conceptualized and supervised the project, acquired funding, curated and analyzed data, developed the underlying methodology, implemented code for data processing, analysis and training machine learning models (regular and graph-convolutional neural networks), validated and visualized the obtained results, as well as wrote the manuscript. D. P. implemented code for training machine learning models (molecular transformer), visualized the corresponding results and contributed toward the writing of the manuscript. W. H. G. and G. K. H. M. supervised the project, as well as reviewed and edited the manuscript.

## Conflicts of interest

There are no conflicts to declare.

## Supplementary Material
